# Postoperative pain after joint replacement surgery: A randomized controlled trial of transcutaneous auricular vagus nerve stimulation and the role of depression and anxiety

**DOI:** 10.1007/s00132-026-04784-2

**Published:** 2026-02-24

**Authors:** Lena Puhl, Claudio Christoph, Rosa Butzlaff, Caroline Rometsch, Christoph H. Lohmann, Markus Ullsperger, Florian Junne, Christian Riediger

**Affiliations:** 1 Department of Psychosomatic Medicine and Psychotherapy, University Hospital Magdeburg, Otto von Guericke University Magdeburg, Magdeburg, Germany; 2 Institute of Psychology, University of Hildesheim, Universitätsplatz 1, 31141 Hildesheim, Germany; 3 Department of Psychosomatic Medicine and Psychotherapy, University Hospital Tübingen, Tübingen, Germany; 4 Department of Psychosomatic Medicine and Psychotherapy, Ilm Valley Clinic, Median Rehabilitation Centre Bad Berka, Bad Berka, Germany; 5 Faculty of Medicine, Martin Luther Universität Halle Wittenberg, Halle (Saale), Germany; 6 Department of Orthopaedic Surgery, University Hospital Magdeburg, Otto von Guericke Universität Magdeburg, Magdeburg, Germany; 7 Otto von Guericke Universität Magdeburg, Institute of Psychology, Magdeburg, Germany

**Keywords:** Vagus nerve stimulation, Neuromodulation, Arthroplasty, Mental comorbidity, Pain management, Vagusnervstimulation, Neuromodulation, Endoprothetik, Psychische Komorbidität, Schmerzmanagement

## Abstract

**Background:**

Chronic postoperative pain is a common and often insufficiently treated complication that is closely linked with depressive and anxiety symptoms.

**Objective:**

To investigate whether transcutaneous auricular vagus nerve stimulation (taVNS) reduces postoperative pain and if depressive or anxiety symptoms modulate the treatment effects.

**Methods:**

In a three-arm, double-blind, randomized controlled trial, 44 patients undergoing joint replacement surgery were allocated to an intervention group (*n* = 15), sham group (*n* = 17), or treatment as usual (TAU) control group (*n* = 12). Assessments were conducted at hospital admission (T_0_) and before discharge (T_1_) using the Short-Form McGill Pain Questionnaire (SF-MPQ), the Patient Health Questionnaire‑9 (PHQ-9) and the Generalized Anxiety Disorder 7 (GAD-7). Data were analyzed using mixed-design ANOVA and linear mixed-effects regression models with a Bonferroni correction.

**Results:**

There were no significant main or interaction effects of group, time, or group × time on pain intensity (F(2,18) = 0.04, *p* = 0.959, η2 = 0.003; F(1,18) = 0.89, *p* = 0.358, η2 = 0.015). The PHQ‑9 scores showed trends for group and time effects (F(2,21) = 5.96, *p* = 0.009, η2 = 0.322; F(1,21) = 5.06, *p* = 0.035, η2 = 0.038). The GAD‑7 did not reveal significant effects. Moderation analyses did not indicate any significant effects of depressive or anxiety symptoms on pain change and combined moderation was not significant either.

**Conclusion:**

In the present study, taVNS did not reduce postoperative pain and showed no moderating effects of depression or anxiety. Standardization of stimulation parameters, inclusion of psychological and somatic comorbidities and longer high-frequency follow-up periods are needed for future studies.

**Graphic abstract:**

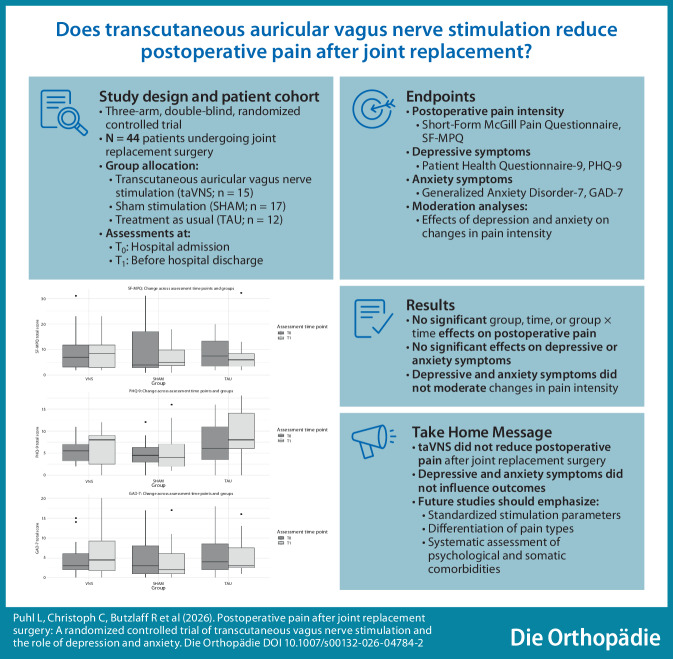

## Abstract (de)

**Hintergrund:**

Chronische postoperative Schmerzen sind eine häufige und oft unzureichend behandelte Komplikation, die eng mit Depressions- und Angstsymptomen verbunden ist.

**Ziel:**

Es sollte untersucht werden, ob die transkutane aurikuläre Vagusnervstimulation (taVNS) postoperative Schmerzen lindert und ob Depressions- oder Angstsymptome die Wirkung der Therapie beeinflussen.

**Methoden:**

In einer dreiarmigen, doppelblinden, randomisiert-kontrollierten Studie wurden 44 Patienten, die sich einer Gelenkersatzoperation unterzogen, einer Interventionsgruppe (*n* = 15), einer Scheingruppe (*n* = 17) oder einer Kontrollgruppe (*n* = 12) zugewiesen. Die Bewertungen wurden bei der Aufnahme ins Krankenhaus (T0) und vor der Entlassung (T1) unter Verwendung des Short-Form McGill Pain Questionnaire (SF-MPQ), des Patient Health Questionnaire‑9 (PHQ-9) und des Generalized Anxiety Disorder 7 (GAD-7) durchgeführt. Die Daten wurden unter Verwendung von Varianzanalysen (ANOVAs) mit gemischtem Design und linearen Regressionsmodellen mit gemischten Effekten und Bonferroni-Korrektur analysiert.

**Ergebnisse:**

Es gab keine signifikanten Haupt- oder Wechselwirkungen von Gruppe, Zeit oder Gruppe × Zeit auf die Schmerzintensität (F(2,18) = 0,04, *p* = 0,959, η2 = 0,003; F(1,18) = 0,89, *p* = 0,358, η2 = 0,015). Die PHQ-9-Werte zeigten Tendenzen für Gruppen- und Zeiteffekte (F(2,21) = 5,96, *p* = 0,009, η2 = 0,322; F(1,21) = 5,06, *p* = 0,035, η2 = 0,038). Der GAD‑7 ergab keine signifikanten Effekte. Moderationsanalysen zeigten keine signifikanten Auswirkungen von Depressions- oder Angstsymptomen auf die Schmerzveränderung, und auch die kombinierte Moderation war nicht signifikant.

**Schlussfolgerung:**

In der vorliegenden Studie reduzierte taVNS die postoperativen Schmerzen nicht und zeigte keine moderierenden Effekte von Depressionen oder Angstzuständen. Für zukünftige Studien sind eine Standardisierung der Stimulationsparameter, die Einbeziehung psychologischer und somatischer Komorbiditäten sowie längere, hochfrequente Nachbeobachtungszeiträume erforderlich.

## Short introduction

Chronic pain impairs physical functioning, mental health, and social participation and is often associated with depression and anxiety. Current postoperative pain management is frequently insufficient to address these interactions, leaving a treatment gap for patients with psychological comorbidities. Therefore, innovative approaches such as transcutaneous auricular vagus nerve stimulation (taVNS) are gaining in relevance.

## Introduction

Chronic pain is defined by the International Association for the Study of Pain (IASP) as an unpleasant sensory and emotional experience associated with actual or potential tissue damage that persists or recurs for more than 3 months [27]. In Germany, approximately 15% of adults suffer from chronic pain [24] with a prevalence of chronic postoperative pain of 12% [9]. Nevertheless, chronic postoperative pain often remains unrecognized and may be insufficiently managed [25].

The 11th revision of the International Classification of Diseases (ICD-11) differentiates chronic primary from chronic secondary pain syndromes [30]. Chronic secondary pain syndromes are directly attributable to an underlying somatic condition and include chronic postoperative and posttraumatic pain [27]. According to the IASP, chronic postoperative pain is defined as a condition following surgical interventions and persisting for at least 3 months beyond expected healing [27]. Given its high prevalence and clinical relevance, postoperative pain represents an important focus for neuromodulative approaches, particularly with respect to its potential transition into a chronic pain condition.

Patients with chronic pain show higher rates of anxiety and depressive disorders compared to healthy populations and bidirectional reinforcement of symptoms [1, 13]. They experience higher postoperative pain intensity [10], increased use of opioids [6] and substantial functional impairment [29].

Current postoperative pain management mainly focuses on pharmacological approaches, while psychological comorbidities are often neglected. Due to this fact, nonpharmacological, neuromodulatory approaches, such as transcutaneous auricular vagus nerve stimulation (taVNS) are gaining clinical interest. The use of taVNS has demonstrated analgesic potential across various pain and psychiatric conditions [5, 28]. This study investigated the effects of taVNS on postoperative pain following joint replacement surgery, with special consideration of comorbid depressive and anxiety symptoms.

## Methods

### Sample

The study included 44 patients (≥ 18 years) scheduled for elective total joint replacement of the hip, knee, shoulder or ankle at the Department of Orthopaedics, University Hospital Magdeburg, Germany. Exclusion criteria comprised cardiac arrhythmia, implanted pacemakers, ear injuries, and severe psychiatric disorders.

Informed consent was obtained from all patients prior to inclusion in the study. The study was conducted in accordance with the Declaration of Helsinki [33] and approved by the Ethics Committee of Otto von Guericke University Magdeburg (Approval No. 85/24).

### Procedure

This three-arm, double-blind, randomized controlled trial recruited participants during preoperative consultations. After screening, baseline data were collected at hospital admission (T_0_), followed by random assignment to one of three conditions: intervention (VNS), sham stimulation (SHAM), or treatment as usual (TAU).

**Fig. 1:**
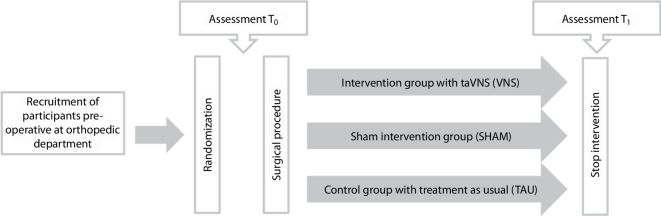
Illustration of the three-arm, randomized controlled study design evaluating the effectiveness of vagus nerve stimulation in patients from the orthopedic department, with allocation to intervention (VNS), sham intervention (SHAM) and control groups (TAU) and data collection before (T_0_) and after (T_1_) the intervention

Following surgery participants in the VNS and SHAM groups received the Nurosym™ (Parasym Ltd., London, United Kingdom) stimulation device [21], targeting the auricular branch of the vagus nerve. The device was applied 2 to 3 times daily for 40–60 min over 3–5 days. Stimulation parameters were set at 20 Hz and 200 μs; participants individually adjusted intensity levels for comfort. The sham device was visually identical but inactive. The control group received standard postoperative care, including analgesia and physiotherapy. A follow-up assessment was conducted before discharge (T_1_) (see Fig. 1).

### Measures

Short-Form McGill Pain Questionnaire (SF-MPQ): assesses sensory and affective pain dimensions on a 0–3 scale across 15 items [20]. Additionally, current pain intensity is rated via a visual analog scale (VAS, 0–100) and an overall pain experience scale (0–5).

Patient Health Questionnaire‑9 (PHQ-9): measures depressive symptom severity on a 0–3 scale across 9 items, with established cut-offs for mild to severe depression [15, 19].

Generalized Anxiety Disorder‑7 (GAD-7): evaluates anxiety severity on a 0–3 scale across 7 items, with cut-offs for mild, moderate and severe anxiety [26].

### Statistical analysis

Data analysis was conducted using RStudio (v2024.12.1 + 563; Posit Software, PBC, Boston, MA, USA) and R (v4.3.3; R Foundation for Statistical Computing, Vienna, Austria [23]). Mixed-design ANOVAs were performed using *anova_test* from the rstatix package (v0.7.2; [14]) and moderation analyses via linear mixed-effects models using *lmer* from the lmerTest package (v3.1.3; [17]). Descriptive statistics were computed for demographic variables. Group and time effects were evaluated with mixed-design ANOVA reporting generalized effect sizes (η^2^g (generalized eta squared); [2]).

Moderation analyses examined whether baseline PHQ‑9 and GAD‑7 scores moderated the relationship between group allocation and changes in pain intensity and quality, testing two, three and four-way interactions. Bonferroni correction [4] was applied (α = 0.05/57 ≈ 0.000877). Missing data were handled by listwise deletion.

## Results

Of the 44 enrolled participants (VNS *n* = 15; SHAM *n* = 17; TAU *n* = 12), 37 completed follow-up (VNS *n* = 12; SHAM *n* = 13; TAU *n* = 12). The mean age was 65.3 years (*SD* = 10.04; range 39–84 years), with 52.3% female. Hip (*n* *=* 17) and knee replacements (*n* *=* 19) predominated.

Median SF-MPQ pain scores remained unchanged (T_0_ median = 6.0; T_1_ median = 6.0). No significant group, time, or interaction effects were found, PHQ‑9 medians increased slightly (T_0_ = 5.0; T_1_ = 6.0) and GAD‑7 medians decreased (T_0_ = 4.0; T_1_ = 3.0); however, none reached significance after statistical correction. Depression and anxiety did not moderate pain change; combined moderation was also not significant.

### Sample description

**Fig. 2:**
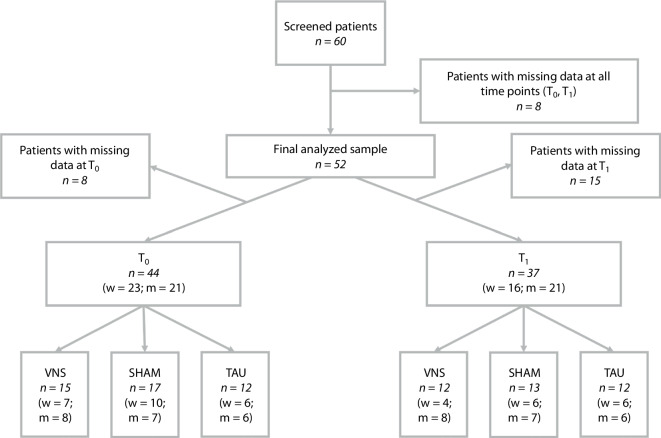
Flowchart illustrating the composition of the sample in the vagus nerve stimulation study, showing screened patients (*n* = 60), exclusions due to missing data and the final analyzed sample before (T0, *n* = 44) and after (T1, *n* = 37) the intervention, including allocation to the intervention (VNS), sham intervention (SHAM) and control group (TAU)

At baseline (T_0_), the study sample consisted of 44 patients: 15 in the VNS group, 17 in the SHAM group, and 12 in the TAU group. At discharge (T_1_), data from 37 patients were available for analysis (VNS: *n* *=* 12; SHAM: *n* *=* 13; TAU: *n* *=* 12) (see Fig. 2).

Participants had a mean age of 65.3 years (*SD* = 10.04; 39–84 years), with a balanced gender distribution (23 female = 52.27%; 21 male = 47.73%). Most patients underwent hip (*n* = 17) or knee replacement (*n* = 19) (see Table 1 for further details).

**Table 1:** Sociodemographic and clinical characteristics of patients undergoing vagus nerve stimulation before (T_0_) and after (T_1_) the intervention (*n* *=* 13)

		T_0_ (*n* = 44)	T_1_ (*n* = 37)
Age (years)	Mean (SD)	65.3 (10.04)	65.35 (10.01)
Sex	Male	21 (47.73%)	21 (56.76%)
	Female	23 (52.27%)	16 (43.24%)
Surgical indication	Hip replacement	17 (38.64%)	14 (37.84%)
	Knee replacement	19 (43.18%)	16 (43.24%)
	Ankle replacement	7 (15.91%)	6 (16.22%)
	Shoulder replacement	1 (2.77%)	1 (2.70%)
**Additional sociodemographic data (*n*** **=** **13)**			
Marital status	Married	12 (92.31%)	
	Divorced	1 (7.69%)	
Religion	None	10 (76.92%)	
	Christianity	3 (23.08%)	
Migration background	None	12 (92.31%)	
	Present	1 (7.69%)	
Highest educational degrees	Secondary school diploma	1 (7.69%)	
	(Advanced) high school diploma	5 (38.46%)	
	Vocational training	1 (7.69%)	
	University degree	5 (38.46%)	
	Doctorate/habilitation	1 (7.69%)	
Employment status	Full-time employed	4 (30.77%)	
	Unemployed	1 (7.69%)	
	Retired	8 (61.54%)	
Monthly net income (*n* = 12)	< 1000 €	2 (16.67%)	
	1000–1999 €	5 (41.67%)	
	2000–2999 €	3 (25.00%)	
	3000–3999 €	1 (8.33%)	
	≥ 4000 €	1 (8.33%)	
Previous surgeries	None	1 (7.69%)	
	Present	12 (92.31%)	
Number of previous surgeries	Mean (SD)	4.58 (3.47)	
Regular use of analgesics	No	7 (53.85%)	
	Yes	6 (46.15%)	
Regular use of psychopharmaceuticals	No	13 (100.00%)	
	Yes	0 (0.00%)	
Duration of preoperative pain (months)	Mean (SD)	36.38 (49.71)	
History of chronic pain	No	5 (38.46%)	
	Yes	8 (61.54%)	
Previous pain therapy experience	No	13 (100.00%)	
	Yes	0 (0.00%)	
Previous psychotherapy experience	No	13 (100.00%)	
	Yes	0 (0.00%)	
Previous neuromodulation experience	No	13 (100.00%)	
	Yes	0 (0.00%)	

### Effect of the intervention on pain symptoms

**Fig. 3:**
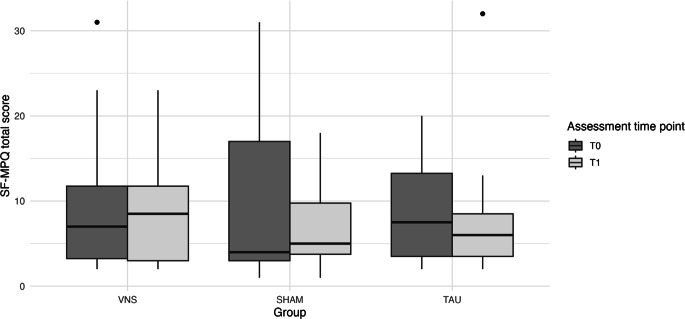
Changes in pain intensity and pain quality, measured using the Short-Form McGill Pain Questionnaire (SF-MPQ), before (T_0_) and after (T_1_) the intervention across the study groups: the intervention group receiving vagus nerve stimulation (VNS), the sham intervention group (SHAM), and the control group (TAU)

At T_0_, the median pain score was 6.0 (Q1 = 3.0; Q3 = 13.5; range = 1–31), and at T_1_, the median remained 6.0 (Q1 = 3.0; Q3 = 10.5; range *=* 1–32). The mixed-design ANOVA revealed no significant main effects of group (*F*_*(2, 18)*_ *=* *0.04, p* *=* *0.959, η*^*2*^ *=* *0.003*) or time (*F(*_*1, 18*_*)* *=* *0.89, p* *=* *0.358, η*^*2*^ *=* *0.015*). The group × time interaction was also nonsignificant (*F(*_*2, 18*_*)* *=* *0.41, p* *=* *0.668, η*^*2*^ *=* *0.014*) (see Fig. 3).

### Effect of the intervention on depressive symptoms

**Fig. 4:**
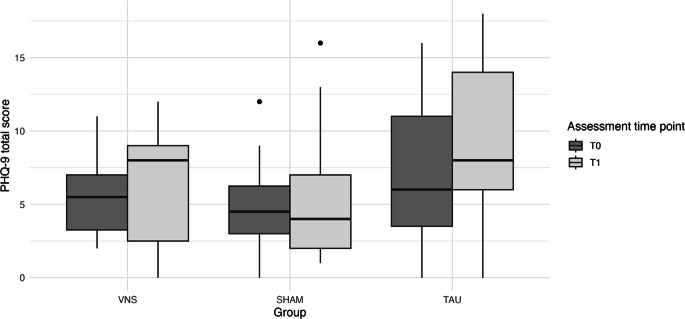
Change in depressive symptoms, assessed using the Patient Health Questionnaire‑9 (PHQ-9), before (T_0_) and after (T_1_) the intervention across study groups: intervention group with vagus nerve stimulation (VNS), sham intervention group (SHAM), and control group (TAU)

Median PHQ‑9 scores increased from 5.0 (Q1 = 3.0;Q3 = 7.0; range = 0–16) at T_0_ to 6.0 (Q1 = 3.5; Q3 = 9.5; range = 0–18) at T_1_ (see Table 2). Mixed ANOVA revealed a main effect of group (*F*_*(2, 21)*_ *=* *5.96, p* *=* *0.009, η*^*2*^ *=* *0.322*) and time (*F*_*(1, 21)*_ *=* *5.06, p* *=* *0.035, η*^*2*^ *=* *0.038*); however, both failed to reach the Bonferroni-adjusted significance threshold (*p* *<* *0.00088*). The interaction effect between group and time was nonsignificant (*F*_*(2, 21)*_ *=* *0.90, p* *=* *0.420, η*^*2*^ *=* *0.014*) (see Fig. 4).

**Table 2:** Severity of depressive symptoms, assessed with the Patient Health Questionnaire‑9 (PHQ-9), and severity of anxiety symptoms, assessed with the Generalized Anxiety Disorder‑7 (GAD-7), in patients before (T_0_) and after (T_1_) the intervention

	Number at T_0_ (*n*, %)	Number at T_1_ (*n*, %)
**PHQ-9: Severity of depressive symptoms**		
*No indication* (< 5 points)	17 (41.5%)	12 (34.3%)
*Mild* (5–9 points)	19 (46.3%)	14 (40.0%)
*Moderate* (10–14 points)	4 (9.8%)	5 (14.3%)
*Moderately severe *(15–19 points)	1 (2.4%)	4 (11.4%)
*Severe* (20–27 points)	0 (0.0%)	0 (0.0%)
**GAD-7: Severity of anxiety symptoms**		
*No indication* (< 5 points)	25 (58.1%)	20 (55.6%)
*Mild *(5–9 points)	10 (23.3%)	8 (22.2%)
*Moderate *(10–14 points)	4 (9.3%)	5 (13.9%)
*Moderately severe* (15–19 points)	4 (9.3%)	3 (8.3%)

### Effect of the intervention on anxiety symptoms

**Fig. 5:**
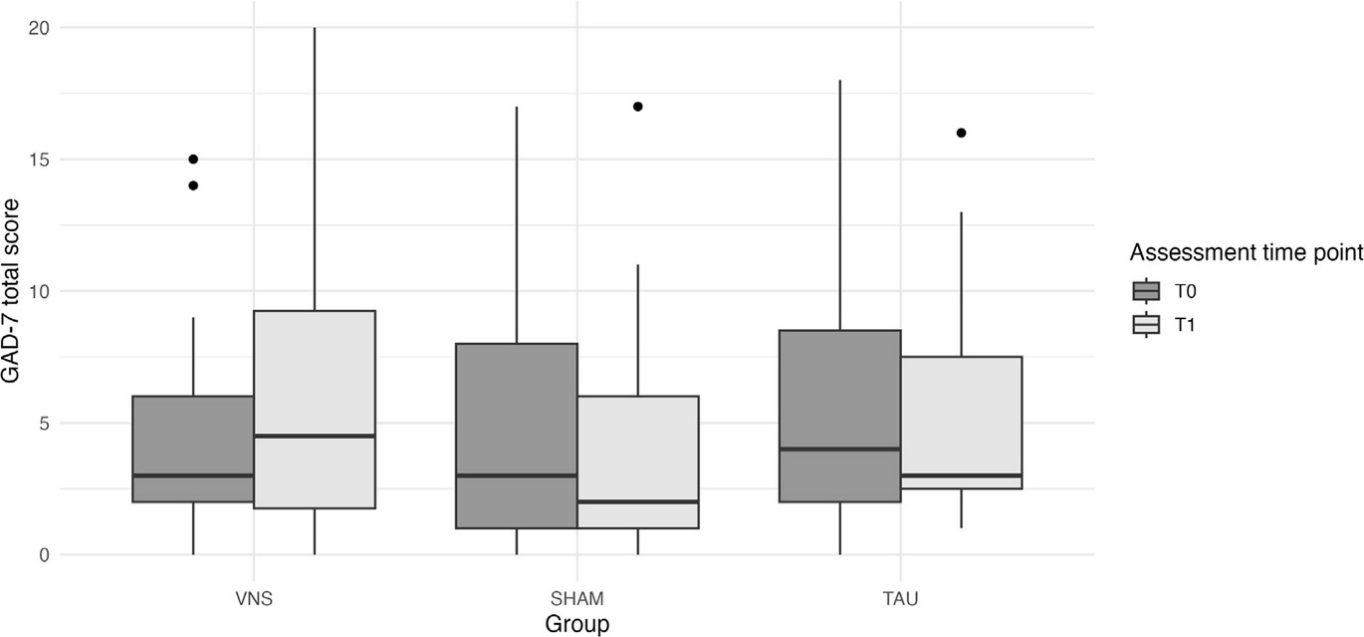
Change in anxiety symptoms as measured by the Generalized Anxiety Disorder‑7 (GAD-7) before (T_0_) and after (T_1_) the intervention across study groups: intervention group receiving vagus nerve stimulation (VNS), sham intervention group (SHAM), and control group (TAU)

Median GAD‑7 scores were 4.0 (Q1 = 2.0; Q3 = 8.0; range = 0–18) at T_0_ and 3.0 (Q1 = 1.0; Q3 = 9.0; range = 0–20) at T_1_ (see Table 2). No significant main effects were found for group (*F*_*(2, 24)*_ *=* *0.25, p* *=* *0.780, η*^*2*^ *=* *0.019*) or time (*F*_*(1, 24)*_* <* *0.01, p* *=* *0.983, η*^*2*^* <* *0.001*), and the group × time interaction was also nonsignificant (*F*_*(2, 24)*_ *=* *0.44, p* *=* *0.650, η*^*2*^ *=* *0.003*) (see Fig. 5).

### Moderation analyses: depression and anxiety as moderators of pain change

#### Depressive symptoms as moderator

No significant effects were observed for time, group, or baseline PHQ‑9 score. Two and three-way interactions were also not significant (see Table 3).

**Table 3:** Results of the linear mixed model for the Patient Health Questionnaire‑9 (PHQ-9) total score as a moderator, including regression coefficients (B), standard errors (SE), degrees of freedom (df), t‑values and *p*-values for main and interaction effects before (T_0_) and after (T_1_) the intervention across study groups: vagus nerve stimulation group (VNS), sham intervention group (SHAM), and control group (TAU)

Effect/predictor	B	SE	df	t	*p*
(Intercept)	3.41	6.58	44.99	0.52	0.607
Assessment time point T_1_	5.05	8.35	36.53	0.61	0.549
Group SHAM	0.72	8.27	45.00	0.09	0.931
Group TAU	2.93	7.66	44.98	0.38	0.704
PHQ‑9 total score at T_0_ (PHQ_T_0_)	1.23	1.07	44.95	1.15	0.256
Assessment time point T_1_ and group SHAM	−5.82	11.17	32.76	−0.52	0.606
Assessment time point T_1_ and group TAU	−11.52	10.67	32.98	−1.08	0.288
Assessment time point T_1_ and PHQ_T_0_	−0.94	1.31	33.20	−0.72	0.477
Group SHAM and PHQ_T_0_	−0.60	1.34	44.99	−0.45	0.655
Group TAU and PHQ_T_0_	−0.94	1.16	44.99	−0.81	0.422
Assessment time point T_1_, group SHAM and PHQ_T_0_	1.02	1.92	44.99	0.53	0.600
Assessment time point T_1_, group TAU and PHQ_T_0_	1.76	1.46	31.55	1.21	0.237

#### Anxiety symptoms as moderator

Similarly, there were no significant main effects for time or group. The GAD‑7 total did not reach the Bonferroni-adjusted significance level. Interaction terms were also not significant (see Table 4).

**Table 4:** Results of the linear mixed model for the Generalized Anxiety Disorder‑7 (GAD-7) total score as a moderator, including regression coefficients (B), standard errors (SE), degrees of freedom (df), t‑values, and *p*-values for main and interaction effects before (T_0_) and after (T_1_) the intervention across study groups: vagus nerve stimulation group (VNS), sham intervention group (SHAM), and control group (TAU)

Effect/predictor	B	SE	df	t	*p*
(Intercept)	4.94	3.23	48.76	1.53	0.132
Assessment time point T_1_	2.59	4.24	34.13	0.61	0.544
Group SHAM	−0.32	4.31	48.56	−0.08	0.941
Group TAU	−0.30	4.43	48.16	−0.07	0.946
GAD‑7 total score at T_0_ (GAD_T_0_)	1.00	0.44	48.30	2.29	0.026
Assessment time point T_1_ and group SHAM	−1.50	5.65	31.36	−0.27	0.793
Assessment time point T_1_ and group TAU	7.12	5.98	29.69	1.19	0.244
Assessment time point T_1_ and GAD_T_0_	−0.70	0.53	25.82	−1.33	0.197
Group SHAM and GAD_T_0_	−0.41	0.60	48.38	−0.69	0.493
Group TAU and GAD_T_0_	−0.39	0.57	47.88	−0.70	0.489
Assessment time point T_1_, group SHAM and GAD_T_0_	0.28	0.73	25.57	0.38	0.706
Assessment time point T_1_, group TAU and GAD_T_0_	−0.43	0.69	24.29	−0.62	0.540

#### Combined moderation by depressive and anxiety symptoms

The combined moderation model (baseline PHQ‑9 and GAD-7) revealed no significant main effects for time, group, PHQ‑9, or GAD‑7. No significant two, three, or four-way interactions were observed (see Table 5).

**Table 5:** Results of the linear mixed model with combined moderation by the total scores of the Patient Health Questionnaire‑9 (PHQ-9) and the Generalized Anxiety Disorder‑7 (GAD-7), including regression coefficients (B), standard errors (SE), degrees of freedom (df), t‑values, and *p*-values for main and interaction effects before (T_0_) and after (T_1_) the intervention across study groups: vagus nerve stimulation group (VNS), sham intervention group (SHAM), and control group (TAU)

Effect/predictor	B	SE	df	t	*p*
(Intercept)	16.44	7.05	32.77	2.33	0.026
Assessment time point T_1_	−10.34	7.63	14.40	−1.36	0.196
Group SHAM	−16.59	9.32	32.98	−1.78	0.084
Group TAU	−12.14	8.09	33.00	−1.50	0.143
PHQ‑9 total score at T_0_ (PHQ_T_0_)	−2.00	1.23	33.00	−1.63	0.113
GAD‑7 total score at T_0_ (GAD_T_0_)	−2.65	2.62	32.92	−1.01	0.319
Assessment time point T_1_ and group SHAM	−0.33	11.84	16.75	−0.03	0.978
Assessment time point T_1_ and group TAU	5.82	9.31	13.93	0.63	0.542
Assessment time point T_1_and PHQ_T_0_	1.97	1.40	18.63	1.40	0.177
Group SHAM and PHQ_T_0_	2.83	1.81	32.50	1.56	0.128
Group TAU and PHQ_T_0_	1.96	1.32	32.96	1.49	0.147
Assessment time point T_1_ and GAD_T_0_	3.91	2.68	30.35	1.46	0.155
Group SHAM and GAD_T_0_	4.78	2.88	32.93	1.66	0.107
Group TAU and GAD_T_0_	3.27	2.71	32.86	1.21	0.237
PHQ_T_0_ and GAD_T_0_	0.54	0.30	32.92	1.79	0.083
Assessment time point T_1_, group SHAM and PHQ_T_0_	2.22	3.13	24.11	0.71	0.486
Assessment time point T_1_, group TAU aand PHQ_T_0_	0.14	1.51	17.42	0.10	0.926
Assessment time point T_1_, group SHAM and GAD_T_0_	−4.90	3.03	29.65	−1.62	0.117
Assessment time point T_1_, group TAU and GAD_T_0_	−4.22	2.79	28.82	−1.51	0.142
Assessment time point T_1_, PHQ_T_0_ and GAD_T_0_	−0.60	0.30	25.15	−1.96	0.062
Group SHAM, PHQ_T_0_ and GAD_T_0_	−0.74	0.34	32.87	−2.20	0.035
Group TAU, PHQ_T_0_ and GAD_T_0_	−0.53	0.31	32.88	1.73	0.092
Assessment time point T_1_, group SHAM, PHQ_T_0_ and GAD_T_0_	0.46	0.35	21.84	1.32	0.201
Assessment time point T_1_, group TAU, PHQ_T_0_ and GAD_T_0_	0.46	0.31	24.17	1.48	0.151

## Discussion

In this three-arm randomized controlled trial, transcutaneous auricular vagus nerve stimulation (taVNS) showed stable pain intensity over time, with a slight increase in depressive symptoms and a decrease in anxiety symptoms without any significant statistical differences between groups or time points. Neither depressive nor anxiety symptoms influenced changes in postoperative pain; however, the heterogeneity of baseline pain intensity in this sample may have influenced the treatment response. Existing evidence suggests that taVNS efficacy varies by pain type [7], with promising results for knee arthroplasty [12, 22] and fibromyalgia [16]. Prior studies typically measured pain immediately before and after stimulation sessions [16, 22] or across several postoperative days [12]. Others used extended follow-up intervals (4–20 weeks) [18, 31]. Earlier and direct postoperative application might favorably influence acute pain processes but the patient condition often limits feasibility.

Although taVNS treatment in postoperative pain was already investigated in various studies< there is still no standardized protocol for patient-reported outcome measures as well as the implementation procedure. Therefore, it is difficult to report comparable data.

For example, psychological comorbidities were assessed using self-report questionnaires (PHQ‑9, GAD-7), whereas other studies employed clinician-rated or alternative self-report tools such as the Beck Depression Inventory [12, 16], Montgomery-Åsberg Depression Rating Scale [18], Beck Anxiety Inventory [8, 12] and Hamilton Anxiety Scale [18]. The Amsterdam Preoperative Anxiety and Information Scale [22] further captures surgery-related anxiety and information needs [3]. This diversity of instruments highlights the lack of standardized psychological assessment.

Moreover, there are reports from other studies that the intervention duration commonly spanned 4–8 weeks, with 30-min sessions once or twice daily [11, 12, 16, 18, 31]. Thus, future studies could provide data on whether more frequent and longer term assessments may distinguish acute from sustained effects of this treatment.

Furthermore, the stimulation protocol used here applied left-sided taVNS at 20 Hz and 200 μs pulse width, whereas previous studies varied in stimulation site (unilateral vs. bilateral) and electrode placement [16, 22]. Current intensities also differed, ranging from fixed to individually adjusted thresholds [12, 18]. The relationship between stimulation parameters and clinical efficacy remains unclear, underscoring the need for standardized stimulation protocols [28].

In addition, pain assessment in this study relied on the SF-MPQ, whereas many comparable studies used the VAS [12, 16, 22], which is suitable for standardized, daily pain tracking. Combining qualitative (SF-MPQ) and quantitative (VAS) measures may enhance sensitivity in future trials.

Moreover, sample size and age distribution are important influencing factors in this study. No age restrictions were applied here, whereas other studies limited inclusion to 18–65 years [18, 31] to reduce heterogeneity and comorbidity-related variance [16].

Additionally, some studies assessed quality of life [16, 18] and sleep quality [12, 18], parameters that should be considered in future research.

With respect to methodological aspects, this study followed a randomized controlled design with three conditions, representing a robust design relative to open-label or single-blind studies [11, 16]; however, the short recruitment and intervention period may have masked treatment effects. Other trials report recruitment spanning 14 months to several years [22, 31].

From an orthopedic perspective, the present findings suggest that taVNS, as applied in the early postoperative phase (3–5 days), does not demonstrate a measurable additional benefit over standard postoperative care with respect to pain reduction.

Overall, the current literature reveals substantial methodological heterogeneity, which limits comparability and hinders conclusive evaluation of taVNS efficacy. Future studies should therefore adopt standardized, harmonized protocols for stimulation and assessment, with particular emphasis on clinically relevant orthopedic outcomes and postoperative recovery parameters to give a recommendation for the implementation of taVNS.

## Limitations

The intervention period may have been too short to induce measurable changes in pain intensity, and the restricted follow-up limited the assessment of medium-term or long-term effects. As the observation period ended at hospital discharge, no final conclusions can be drawn regarding chronic postoperative pain. In addition, the relatively small sample size resulted in limited statistical power, particularly with respect to the moderation analyses.

Blinding could be improved and minor sensory sensations such as tingling might unblind participants, as observed in other studies [22, 31]. Implementing minimal sensory stimulation could enhance blinding reliability.

Clinical heterogeneity resulting from the inclusion of different joint replacement procedures may have influenced the results. No stratified or subgroup analyses by joint type were performed. From an orthopedic perspective, procedure-specific effects would be of particular relevance in future investigations.

While self-report assessment instruments enable efficient data collection, they may reduce measurement precision [32]. Future research should include outcome parameters, such as mobility, rehabilitation course, length of hospital stay or opioid consumption.

## Conclusion

This study provides important insights into the use of taVNS in the postoperative context following joint replacement surgery. No significant effects on pain intensity, depressive or anxiety symptoms, and moderating effects of these psychological factors on pain changes were observed; however, the findings contribute substantially to the advancement of taVNS research. The results highlight the importance of standardizing stimulation parameters, using consistent measurement instruments, and collecting closely monitored longitudinal data in future studies. Furthermore, systematic documentation of psychological and somatic comorbidities will improve interpretability. Multicenter studies with larger samples and longer observation periods are required to establish robust evidence for taVNS efficacy and its potential component of integrative postoperative care.

## Practical conclusion

- Based on the present data, taVNS showed no clinically relevant reduction in postoperative pain or psychological symptoms. Given the high heterogeneity of the available evidence, a final recommendation for routine use in early postoperative care following joint replacement surgery cannot be made.
- Future research should focus on standardized stimulation protocols, validated and consistent assessment instruments, and procedure-specific study designs with longer follow-up periods and functional orthopedic outcome measures to further evaluate the efficacy of taVNS as an addition to established postoperative treatment pathways.
- Multicenter trials with larger sample sizes are required before taVNS can be considered as a potential adjunctive component of integrative postoperative orthopedic care.

## Data Availability

Data are available on reasonable request.

## Abbreviations

GAD-7: Generalized Anxiety Disorder-7
ICD-11: International Classification of Diseases, 11th Revision
IASP: International Association for the Study of Pain
PHQ-9: Patient Health Questionnaire-9
SHAM: sham stimulation group
SF-MPQ: Short-Form McGill Pain Questionnaire
TAU: control group (treatment-as-usual)
taVNS: Transcutaneous auricular vagus nerve stimulation
VAS: Visual Analog Scale
VNS: intervention group (vagus nerve stimulation)
